# Adverse childhood experiences among people with schizophrenia at comprehensive specialized hospitals in Bahir Dar, Ethiopia: a comparative study

**DOI:** 10.3389/fpsyt.2024.1387833

**Published:** 2024-05-21

**Authors:** Birhanu Assefa Fentahun, Tilahun Belete Mossie, Rekik Damtew Hailu, Tilahun Bete, Solomon Moges Demeke

**Affiliations:** ^1^ Department of Psychiatry, College of Health and Medical Sciences, Haramaya University, Harar, Ethiopia; ^2^ Department of Psychiatry, College Medical and Health Sciences, Bahir Dar University, Bahir Dar, Ethiopia; ^3^ College of Health Sciences, Woldia University, Woldia, Ethiopia

**Keywords:** schizophrenia, adverse childhood experiences, comparative study, amhara, Ethiopia

## Abstract

**Background:**

People who have encountered adverse childhood experiences (ACEs) are predisposed to developing schizophrenia, experiencing exacerbated symptoms, and facing an elevated risk of disease relapse. It is imperative to evaluate the prevalence of ACEs to comprehend the specific attributes of this condition and enable the implementation of suitable interventions.

**Aims:**

The aim of this study was to assess the prevalence of ACEs and its determinants among people with schizophrenia and the patient attendants in Bahir Dar, Ethiopia.

**Method:**

A comparative cross-sectional study was carried out at the Comprehensive Specialized Hospitals in Bahir Dar, Ethiopia, from April 26 to June 10, 2023. A total of 291 individuals with schizophrenia and 293 individuals from the patient attendants were selected using a systematic random sampling method. A proportional odds model ordinal logistic regression analysis was used to identify the factors associated with ACEs.

**Results:**

The occurrence of at least one ACE among individuals diagnosed with schizophrenia was 69.4%, while patient attendants had a 46.8%, as indicated by the Chi-square test, which showed a significant difference at p <0.05. The study findings indicated that individuals with schizophrenia who have encountered four or more ACEs are more inclined to have lower educational attainment (AOR: 4.69 [1.94 - 11.61]), low resilient coping mechanisms (AOR: 2.07 [1.11 - 3.90]), and poor social support (AOR: 3.93 [2.13 - 7.32]). Conversely, factors such as rural residency, illiteracy, and heightened attachment-related anxiety were found to be notably associated with the patient attendants.

**Conclusion:**

In this study, the substantial prevalence of ACEs emphasized the necessity for ACE screening and the implementation of evidence-based interventions to address and alleviate the overall burden of ACEs.

## Introduction

ACEs are events of a potentially traumatic nature that take place prior to reaching 18 years of age, and have been connected to the primary causes of morbidity and mortality in adulthood (1, 2). These ACEs encompass occurrences involving physical, psychological, and sexual abuse, as well as physical and mental neglect, and dysfunction within the family (3). Individuals with schizophrenia as well as those with other non-psychiatric diseases with severe ACEs, displayed higher levels of comorbidity, necessitated increased medication usage, and required the involvement of a care coordinator compared to individuals with moderate or no ACEs (4–6).

The impact of ACEs extends to diminished well-being, factors influencing quality of life, and substantial impediments in psycho-social functioning that emerge early on in life (7–10). ACEs is associated with unfavorable outcomes, and one possible mechanism through which it can influence results is by causing delays in treatment and a diminished response to antipsychotic interventions (11, 12).

Globally, the prevalence of ACEs among the general population varies from 25% to 85% for individuals with at least one ACE, and from 5.93% to 34.2% for those with four or more ACEs (13–23), while in Africa, percentages range from 58.3% to 72.2% for at least one ACE, and from 14.4% to 39% for three or more ACEs (24, 25). In comparison to the general population, individuals with schizophrenia encountered two to three times more ACEs during childhood (26–30).The occurrence of ACEs among individuals with schizophrenia ranged from 47.2% to 90% for at least one ACE, and from 25% to 52% for four or more ACEs (6, 26–28, 30–32). Although each ACE has detrimental effects on an individual’s health, behavior, and psychological growth, exposure to multiple adverse experiences results in a significantly more damaging impact (33, 34). This implies a dose-response connection, where the risk of adverse effects on physical and mental health exponentially rises with additional ACEs (33, 35). Individuals with schizophrenia who had multiple ACEs histories exhibited more severe symptoms and unfavorable clinical results (4, 36–38). Those with higher levels of ACEs tended to have lower levels of social support, which exhibited a positive correlation with suicide in individuals with schizophrenia, and decreased coping abilities (39–41).

Assessing one’s history of ACEs in adulthood can be advantageous, as it may mitigate the negative repercussions of ACEs on mental health and resilience, and can aid families in breaking the cycle of adversity, subsequently reducing risks and enhancing treatment results (42–46).

Nonetheless, there exists limited evidence regarding ACEs among individuals with schizophrenia in Ethiopia. Therefore, the objective of this research was to ascertain the prevalence of ACEs and their associated factors among individuals with schizophrenia in comparison to the patient attendants. This endeavor could provide valuable insights for healthcare professionals, researchers, and stakeholders interested in working this issue.

## Method

### Study design and setting

A comparative cross-sectional study was conducted from April 26 to June 10, 2023, at Comprehensive Specialized Hospitals in Bahir Dar, Ethiopia.

### Participants

The target participants comprised all individuals with schizophrenia who were hospitalized and receiving outpatient follow-up care, as well as patient attendants (often relatives of the patient) seeking medical treatment from various departments in Hospitals. The study participants included people with schizophrenia who were on follow up and the patient attendants present during the data collection period.

### Inclusion and exclusion criteria

Individuals aged 18 and above with schizophrenia who on follow up, and the patient attendants from medical, surgical, gynecology, obstetric, and pediatric sites in both hospitals (not including psychiatry unit patient relatives)were included in the study. Those who were severely ill or mentally incapacitated were excluded from the study during the data collection period. Furthermore, those who had low cognitive scores in individuals with schizophrenia were excluded using a Mini-Mental State Examination(MMSE) assessment with a total score of less than or equal to 24 on a commonly used cutoff score to define impairment (47). Among the patient attendants, the existence of symptoms of mental illness was assessed using the 18-item Brief Psychiatry Rating Scale (BPRS). Excluded from the study were those who received a BPRS score of 30 or higher, which is considered a baseline for mild mental disorder symptoms (48).

### Sample size estimation and sampling procedure

A formula that is quite general and applies to cross-sectional, case-control, and cohort studies for comparison studies between two proportions (equal sample sizes) was used to determine the sample size (49).


$$n1=n2=\frac{{\left(Z\alpha_{/2}\surd (2pq)+Z\beta \surd (p1q1+p2q2)\right)}^{2}}{(p1\_p2)2}$$

To test the hypothesis P1=P2 versus H1: P1≠P2, for detecting a specific effect: Sample size: n1 = n2; equal ratio: 1:1 = 1; the number of participants required in the two populations.

Effect size: the prevalence of at least one ACE, P1 (cases) (94%), and P2 (healthy participants) (87%), were taken from a previous study (50). P = average proportion exposed (P1 + P2)/2, q = 1-p. Significance Z alpha = power at 95%; statistical power Z beta = power at 80%. Adding 10% of the non-respondent rates, a total of 604 participants were sampled, which means the sample size for each group were 302 in the study.

**Figure 1:**
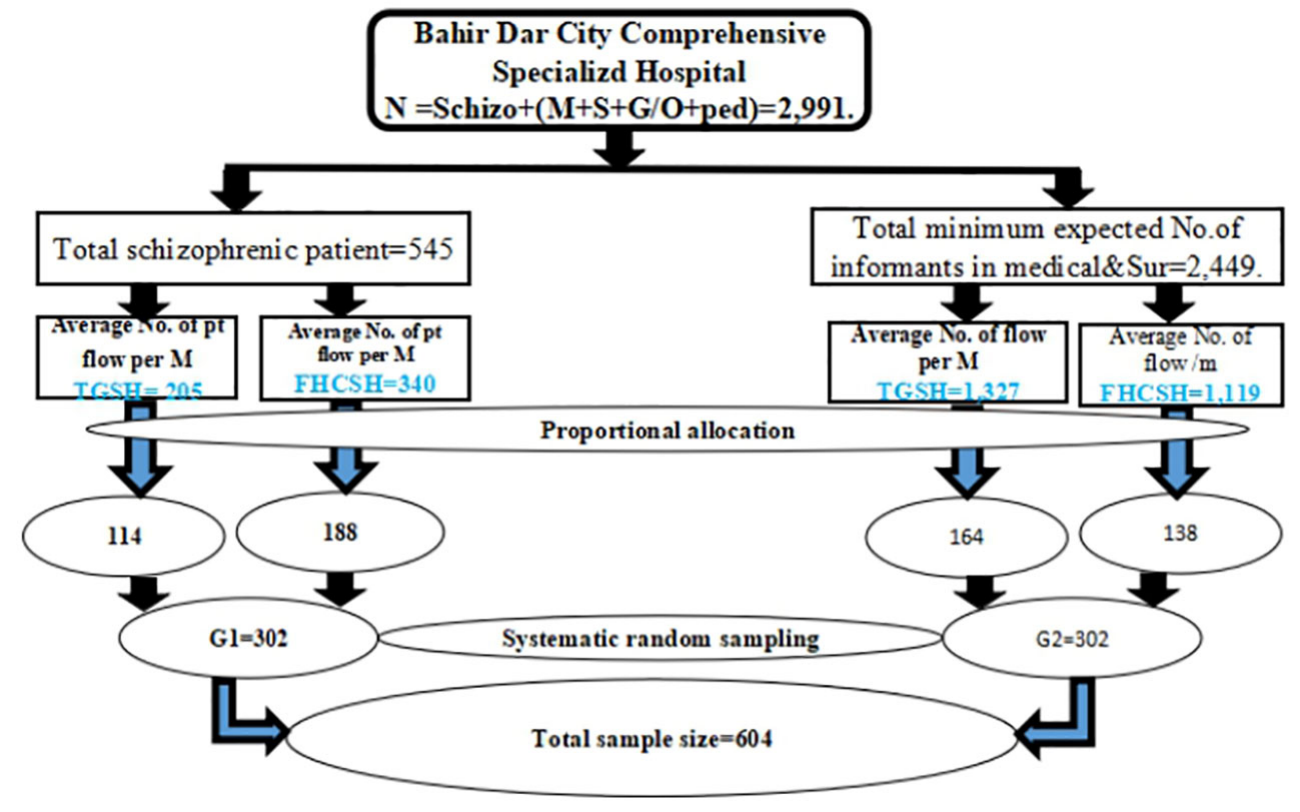
Sampling procedure of selecting study samples from TGSH and FHCSH Bahir Dar, Ethiopia, 2023.

Then, the final sample size was proportionally allocated to TGCSH and FHCSH based on their anticipated monthly case flow (**Figure 1**). A systematic random sampling method was employed, with the patient card list serving as the sampling frame to select the participants. After the sampling interval (K) was determined the first participant was selected through a lottery method, with subsequent participants chosen by adding the calculated K value within each group. In cases where multiple individuals were present in the patient attendants, one was randomly selected.

### Study variables

The study variables included the dependent variable of ACEs (0, 1, 2 and 3, greater than or equal 4). Sociodemographic variables encompassed sex, age, residency, religion, educational status, occupational status, marital status, and ethnicity. Psychosocial factors such as social support, adult attachment, coping strategy, substance-related factors (including alcohol use, khat use, tobacco use), and Clinical factors like suicide behavior and somatic symptoms were also considered.

### Data collection and measurements tool

Structured questionnaire were employed in conducting face-to-face interviews for data collection purposes. The data were acquired by a team of six individuals and supervised by two professionals holding a Bachelor of Science in Psychiatry in each designated study area. The Adverse Childhood Experiences - questionnaire (ACEs-Q), a standardized tool consisting of ten items, was utilized to evaluate the history of ACEs (34). ACEs-Q represents various symptoms of ACEs such as emotional abuse, physical abuse, sexual abuse, emotional neglect, physical neglect, domestic violence, parental separation or death, household substance abuse, household mental illness, and incarcerated household members.

Initially, each ACE was assessed individually. Individuals were classified as exposed to ACEs if they responded affirmatively to one or more questions within a specific category; subsequently, an ACEs score was calculated, assigning one point for each positive response. This scoring system yielded total scores ranging from 0 (no ACEs) to 10 points (exposed to all ACEs). The number of ACEs was grouped into categories of 0, 1, 2 to 3, greater than or equal to 4 ACEs; this was created based on previous studies (34, 51, 52). The ACEs-Q scale has demonstrated strong internal consistency (α=0.86).

#### Experiences in close relationships

Attachment security in an adult population was evaluated using the Experiences in Close Relationships- Relationship Structures (ECR-RS) questionnaire general form (53). ECR-RS general have two scores, one for attachment-related avoidance and the other for attachment-related anxiety. Mean scores were utilized to establish statistical cut-off points for defining high and low anxious or avoidant attachment categories. Scores below the mean on both subscales indicated low anxious or avoidant attachment, while scores equal to or above the mean signified high anxious or avoidant attachment. The ECR-RS scale exhibited excellent internal consistency (α=0.947). The Brief Resilient Coping Scale (BRCS) was applied to gauge individuals’ tendencies to cope effectively with stress. Scores falling within the ranges of four to thirteen, fourteen to sixteen, and seventeen to twenty were categorized as representing low, medium, and high resilient coping, respectively (54). The BRCS, a 4-item measure, displayed good internal consistency (α=0.812). Social support levels were assessed using the Oslo Social Support Scale (OSSS-3), with scores ranging from 3 to 14. Participants were classified as having poor support, intermediate support, or strong social support based on scores of 3 to 8, 9 to 11, and 12 to 14, respectively (55). The OSSS-3 demonstrated acceptable internal consistency (α=0.787).

The assessment of alcohol use extent was carried out utilizing the Alcohol Use Disorder Identification Test (AUDIT) developed by the World Health Organization (WHO). The AUDIT serves as a screening tool to evaluate alcohol use severity levels, categorizing individuals into “no alcohol use,” “social use,” “harmful drinking,” “hazardous drinking,” and “ probable alcohol dependency” in increasing order of severity dependence (56). In the present investigation, the AUDIT exhibited acceptable internal consistency, supported by a Cronbach alpha value of 0.718.Suicide behaviors were measured through the 4-item Suicide Behaviors Questionnaire-Revised (SBQ-R), with individuals scoring seven or more being identified as displaying suicidal behaviors (57). Additionally, the Somatic Symptom Scale-8 (SSS-8) was utilized as a concise 8-item version of the Patient Health Questionnaire-15 (PHQ-15).

The severity categories of SSS-8 were determined based on percentile ranks: ranging from no to minimal (0-3 points), low (4-7 points), medium (8-11 points), high (12-15 points), to very high (16-32 points) somatic symptom burden (58). The SSS-8 is a reliable and valid self-report measure of somatic symptom burden (59). The internal consistency was deemed acceptable with a reliability coefficient of α=0.806.

### Data quality assurance

Data quality assurance was ensured through a meticulous process. Initially, the questionnaire was developed in English, translated into Amharic, and back-translated into English by various language experts to enhance the tool’s reliability and comprehensibility. Subsequently, the tools underwent pretesting with a 5% (31) sample size at FHCSH. Following feedback from the pretest, the final version of the questionnaire was refined. Rigorous training was provided to both data collectors and supervisors, and daily checks on data collection were conducted by the principal investigator and supervisors.

### Data processing and analysis

The data underwent thorough scrutiny for completeness before being exported to Statistical Package for Social Sciences (SPSS) software version 26 for coding and analysis. Utilizing descriptive and inferential statistics, the analysis aimed to describe the findings and explore associations between dependent and independent variables through proportional odds model ordinal logistic regression. Variables with a p-value < 0.25 were included in the final model, which underwent assessments for fitness, goodness of fit and parallel lines. Statistical significance was established at a p-value < 0.05, with the strength of associations estimated through odds ratios with a 95% confidence interval.

### Ethical consideration and consent to participate

Ethical approval was granted by Bahir Dar University College of Medical and Health Science Institutional Review Board (IRB) under protocol number 781/2023, in adherence to local regulations and institutional guidelines. The study adhered to the principles of the Helsinki Declaration on Medical Research Ethics (60). Participants provided written informed consent, and permission was obtained from TGCSH and FHCSH. Participants were assured of their right to withdraw at any time, and information confidentiality and anonymity were strictly maintained.

## Results

### Sociodemographic profile of study participants

In this study, 302 people with schizophrenia and 302 from the patient attendants participated, yielding response rates of 96.35% and 97.02% respectively. Among people with schizophrenia, significant portions were single 103 (35.4%) and aged between 18 and 24 years 115 (39.5%). The majority of people with schizophrenia were female 147 (50.5%) and urban residents 161 (55.3%). In comparison, the patient attendants had a higher proportion of married individuals 179 (60.1%), males 148 (50.5%), and urban residents 151 (51.5%). A statistically significant chi-square test was observed for occupation and marital status, while gender, age, residency, religion, education, and ethnicity did not exhibit significant differences (**Table 1**).

**Table 1:** Sociodemographic information among people with schizophrenia and patient attendant participants included in the study at TGCSH and FHCSH, Bahir Dar, Ethiopia, in 2023.

Variables	Categories	Person with schizophrenia (n=291)		Patient attendants (n=293)		P-value
		Frequency	Percentage	Frequency	Percentage	
Sex of respondents	Male	144	49.5	148	50.5	0.80
	Female	147	50.5	145	49.5	
Age of respondents	18-24	115	39.5	101	34.5	0.25
	25-34	103	35.4	97	33.1	
	35-44	52	17.9	65	22.2	
	45 and above	21	7.2	30	10.2	
Residency	Rural	130	44.7	142	48.5	0.36
	Urban	161	55.3	151	51.5	
Marital status	Single	103	35.4	77	26.3	< 0.005
	married	135	46.4	179	60.1	
	Divorced	33	11.3	23	7.85	
	Widowed	20	6.9	14	4.8	
Religion	Orthodox	230	79.0	241	82.3	0.39
	Protestant	15	5.2	17	5.8	
	Muslim	46	15.8	35	11.9	
Education	Unable to read and write	55	18.9	34	11.6	0.068
	Able to write and read	54	18.6	53	18.1	
	Primary (1-8 grade)	54	18.6	60	20.5	
	Secondary (9-10 grade)	74	25.4	71	24.2	
	Higher education (diploma and above)	54	18.6	75	25.6	
Occupation	House wife	14	4.8	36	12.3	<0.001
	Farming	56	19.2	49	16.7	
	Civil servant	31	10.7	60	20.5	
	Merchant	45	15.5	59	20.1	
	Daily laborer	48	16.5	49	16.7	
	No job	97	33.3	40	13.7	
Ethnicity	Amhara	235	80.8	224	76.5	0.22
	Tigre	12	4.1	7	2.4	
	Oromo	4	1.4	6	2.05	
	Agew	30	10.3	47	16.04	
	other	10	3.4	9	3.07	

Corresponding to the chi **^2^** test for categorical variables, p< 0.05.

### Magnitude of ACEs among people with schizophrenia and the patient attendants

In exploring the nature of ACEs, prevalent ACEs in individuals with schizophrenia included emotional neglect at 38% and emotional abuse at 32%. Conversely, emotional abuse was reported in 24.6% of the patient attendants, while 19.1% experienced situations like having one or no parents, parental separation, or divorce. Sexual abuse emerged as the least common ACEs in both groups. Except for a history of family member incarceration, all other ACEs types were more frequent in individuals with schizophrenia. Statistical analysis through the Chi-square test revealed that household substance abuse, contact sexual abuse, and family member imprisonment did not display significant differences between the two groups (**Table 2**).

**Table 2:** ACEs type among people with schizophrenia and patient attendant participants.

ACEs	Schizophrenic group (n=291) % (CI95%)	Patient attendants (n=293) % (CI95%)	χ2	P
Emotional Abuse	32 (26.1 - 37.5)	24.6 (18.9 - 29.7)	3.93	0.047
Physical Abuse	24.1 (19.2 - 28.8)	15.7 (11.4 - 20.1)	6.40	0.011
Emotional neglect	38.8 (33.3 - 44.3)	15.7 (11.9 - 20)	39.43	< 0.001
Physical neglect	24.1 (19.2 - 28.5)	10.6 (6.9 - 14.3)	18.53	< 0.001
Contact sexual abuse	6.5 (3.9 - 9.6)	3.4 (1.7 - 5.7)	3.00	0.083
Household member treated violently	25.1 (19.7 - 29.6)	13.0 (9.3 - 17.1)	13.92	< 0.001
One / no parents, parental separation / divorce	22.0 (17.1 - 27.0)	19.1 (15 - 24.4)	5.42	0.020
Household Substance Abuse	18.9 (14.8 - 23.7)	11.9 (8.2 - 16.4)	0.742	0.389
Mental Illness in Household	13.4 (9.6 - 17.5)	6.5 (3.8 - 9.2)	7.81	0.005
Incarcerated-household Members	11.3 (8.2 - 15.7)	14.3 (10.6 - 19)	1.17	0.280

Respondents were defined as exposed to an ACE category if they responded “yes” to one or more questions in that category. Chi-square test when comparing patients and patient attendants, P<0.05.

**Figure 2:**
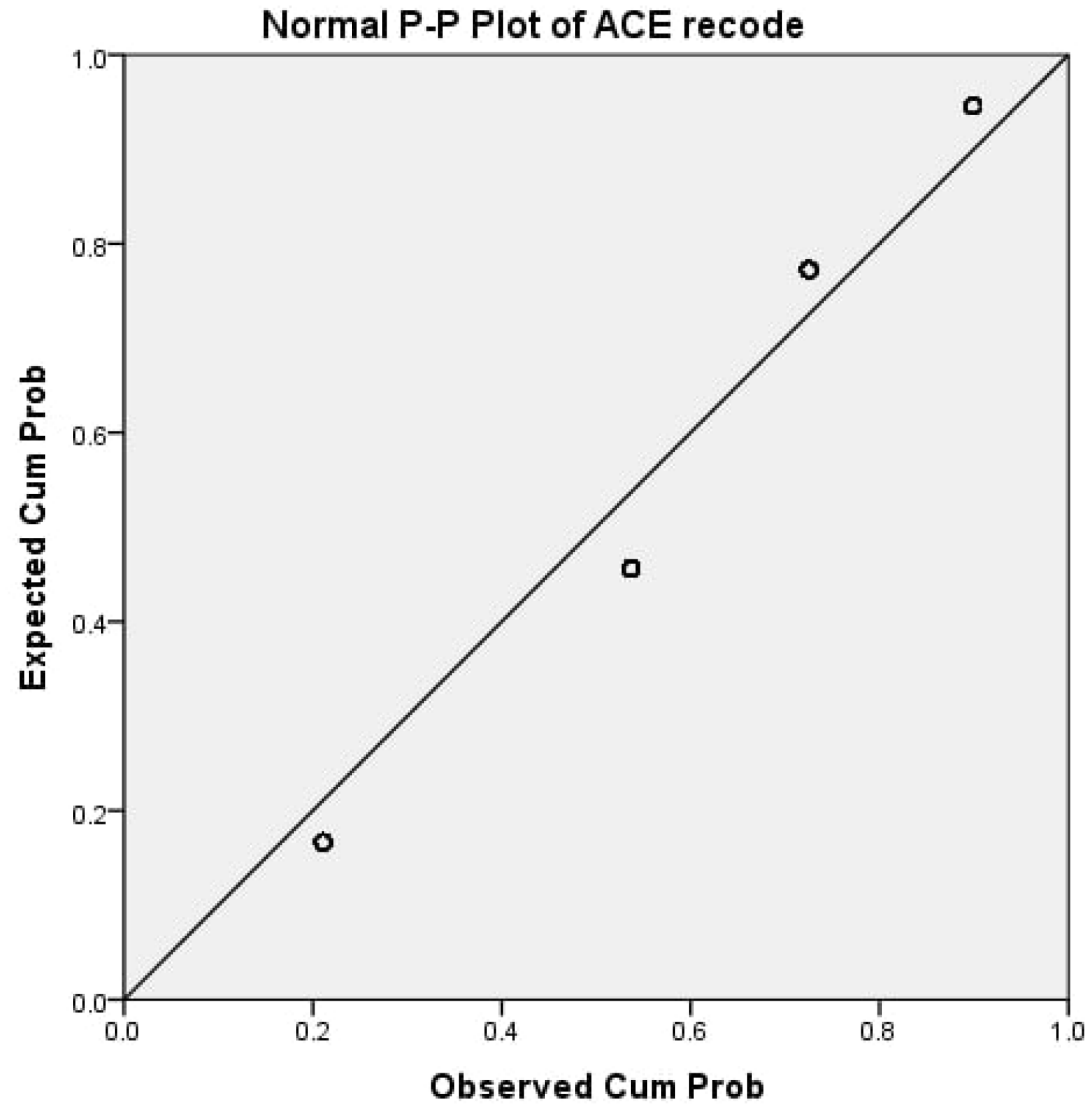
P-P plot of the outcome variable.

**Figure 3:**
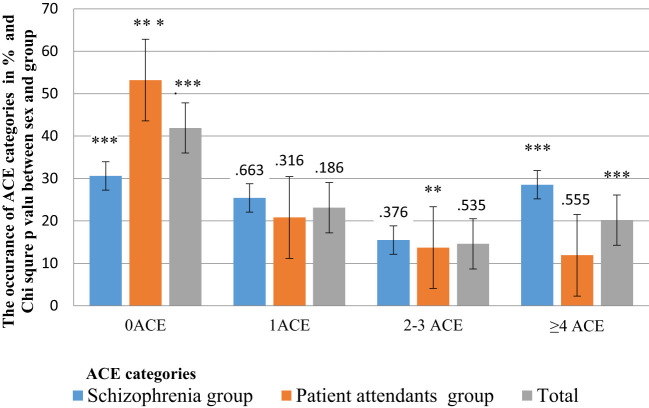
Magnitude of ACEs among people with schizophrenia and the patient attendants. Asterisks denote statistically significant “p” according to the scheme: *p ≤ 0.1, **p ≤ 0.05, ***p ≤ 0.01.

A p-p plot illustrates how the ACEs scores were distributed among all participants (**Figure 2**). Approximately 30.6% of individuals with schizophrenia and 53.2% of the patient attendants did not report any ACEs. On the other hand, 28.5% of those with schizophrenia and 11.9% of the patient attendants encountered four or more ACEs. Moreover, the prevalence of at least one ACE was notably higher among individuals with schizophrenia, with 69.4% reporting ACEs compared to 46.8% in the patient attendants. The statistical significance of this difference was confirmed through the Chi-square test (p < 0.05). When examining the distribution of ACEs scores between the schizophrenia and patient attendants groups, no statistically significant variance was found for individuals with only one type of ACEs or two to three ACEs (**Figure 3**).

#### Health-related risk factors

A considerable proportion, 94 (32.3%) of individuals with schizophrenia and 60 (20.5%) of the patient attendants, exhibited high attachment-related anxiety. Similarly, poor social support was prevalent in 107 (36.8%) of individuals with schizophrenia compared to 43 (14.7%) in the patient attendants. In terms of resilient coping skills, 84 (28.9%) of individuals with schizophrenia and 40 (13.7%) of the patient attendants displayed low levels of resilience (**Table 3**).

**Table 3:** Prevalence of health-related risk factors respect to ACEs score category.

Variables	Categories	ACE Count for people with schizophrenia				Total n=291	ACE Count for the patient attendants				Total n=293	P value
		0ACE	1ACE	2-3ACE	≥4ACE		0ACE	1ACE	2-3ACE	≥4ACE		
Attachment related avoidance	Low (%)	83 (40.3)	65 (31.6)	26 (12.6)	32 (15.5)	206 (70.8)	141 (89.8)	52 (85.2)	18 (45)	17 (48.6)	228 (77.8)	.
	High (%)	6 (7.1)	9 (10.6)	19 (22)	51 (60)	85 (28.2)	16 (10.2)	9 (14.8)	22 (55)	18 (51.4)	65 (22.2)	
Attachment related anxiety	Low	83 (42.1)	56 (28.4)	26 (13.2)	32 (16.2)	197 (67.7)	146 (93)	51 (83.6)	24 (60)	12 (34.3)	233 (79.5)	< 0 .001
	High	6 (6.4)	18 (19.1)	19 (20.2)	51 (54.3)	94 (32.3)	11 (7)	10 (16.4)	16 (40)	23 (65.7)	60 (20.5)	
Brief Resilient Coping	Low resilient cope	13 (14.6)	22 (29.7)	13 (28.9)	36 (43.4)	84 (28.9)	18 (11.5)	5 (8.2)	4 (10)	13 (37.1)	40 (13.7)	< 0 .001
	Medium resilient cope	36 (40.4)	20 (27)	16 (35.6)	22 (26.5)	94 (32.3)	57 (36.3)	22 (36.1)	11 (27.5)	15 (42.9)	105 (35.8)	
	High resilient cope	40 (44.9)	32 (43.2)	16 (35.6)	25 (30.1)	113 (38.8)	82 (52.2)	34 (55.7)	25 (62.5)	7 (20)	148 (50.5)	
Social support	*poor social support*	14 (13.1)	18 (16.8)	24 (22.4)	51 (47.7)	107 (36.8)	19 (12.1)	6 (9.8)	5 (12.5)	13 (37.1)	43 (14.7)	< 0 .001
	*Moderate social support*	13 (21.3)	17 (27.9)	11 (18)	20 (32.8)	61 (21)	71 (45.2)	25 (41)	17 (42.5)	13 (37.1)	126 (43)	
	*Strong social support*	62 (50.4)	39 (31.7)	10 (8.1)	12 (9.8)	123 (42.2)	67 (42.7)	30 (49.2)	18 (45)	9 (25.7)	124 (42.3)	
Alcohol use	Abstainer	32 (36)	21 (28.4)	8 (17.8)	7 (8.4)	68 (23.4)	89 (56.7)	39 (63.9)	14 (35)	11 (31.4)	153 (52.2)	< .001
	Social drinker	30 (33.7)	22 (29.7)	12 (26.7)	12 (14.5)	76 (26.1)	50 (31.8)	21 (34.4)	18 (45)	16 (45.7)	105 (35.8)	
	Hazardous drinker	8 (9)	12 (16.2)	9 (20)	20 (24.1)	49 (16.8)	13 (8.3)	1 (1.6)	6 (15)	5 (14.3)	25 (8.5)	
	Harmful drinker	10 (11.2)	10 (13.5)	7 (15.6)	21 (25.3)	48 (16.8)	5 (3.2)	0	2 (5)	3 (8.6)	10 (3.4)	
	Probable alcohol dependence	9 (10.1)	9 (12.2)	9 (20)	23 (27.7)	50 (17.2)	–	–	–	–		
Former khat use	N0	72 (80.9)	60 (81.1)	31 (68.9)	49 (59)	212 (72.9)	148 (94.3)	53 (86.9)	33 (82.5)	27 (77.1)	261 (89.1)	< 0 .001
	Yes	17 (19.1)	14 (18.9)	14 (31.1)	34 (41)	79 (27.1)	9 (5.7)	8 (13.1)	7 (17.5)	8 (22.9)	32 (10.9)	
Former cigarette use	No	79 (32)	66 (26.7)	38 (15.4)	64 (25.9)	247 (84.9)	155 (98.7)	58 (95.1)	36 (90)	32 (91.4)	281 (95.9)	< 0.001
	Yes	10 (22.7)	8 (18.2)	7 (15.9)	19 (43.2)	44 (15.1)	2 (1.3)	3 (4.9)	4 (10)	3 (8.6)	12 (4.1)	
Current khat use	No	84 (30.3)	72 (26)	41 (14.8)	80 (28.9)	277 (95.2)	150 (95.5)	59 (96.7)	36 (90)	27 (77.1)	272 (92.8)	
	Yes	5 (35.7)	2 (14.3)	4 (28.6)	3 (21.4)	14 (4.8)	7 (4.5)	2 (3.3)	4 (10)	8 (22.9)	21 (7.2)	
Current cigarette use	No	88 (31.2)	71 (25.2)	43 (15.2)	80 (28.4)	282 (96.9)	154 (98.1)	61 (100)	39 (97.5)	29 (82.9)	283 (96.6)	
	Yes	1 (11.1)	3 (33.3)	2 (22.2)	3 (33.3)	9 (3.1)	3 (1.9)	–	1 (2.5)	6 (17.1)	10 (10.4)	
Suicide behavior	No	80 (89.9)	62 (83.8)	30 (66.7)	47 (56.6)	219 (75.3)	149 (94.9)	56 (91.8)	35 (87.5)	30 (85.7)	270 (92.2)	< 0 .001
	Yes	9 (10.1)	12 (16.2)	15 (33.3)	36 (43.4)	72 (24.7)	8 (5.1)	5 (8.2)	5 (12.5)	5 (14.3)	23 (7.8)	
Somatic Symptom	No to minimal	20 (25.5)	24 (32.4)	11 (24.4)	17 (20.5)	72 (24.7)	72 (45.9)	29 (47.5)	13 (32.5)	13 (37.1)	127 (43.3)	< 0 .001
	Low	18 (20.2)	9 (12.2)	14 (31.1)	26 (31.3)	67 (23)	45 (28.7)	17 (27.9)	8 (20)	7 (20)	77 (26.3)	
	Medium	22 (24.7)	15 (20>3	5 (11.1)	10 (12)	52 (17.9)	18 (11.5)	6 (9.6)	12 (30)	7 (20)	43 (14.7)	
	High	19 (21.3)	14 (18.9)	8 (17.8)	14 (16.9)	55 (18.9)	9 (5.7)	6 (9.8)	4 (10)	5 (14.3)	24 (8.2)	
	Very high	10 (11.2)	12 (16.2)	7 (15.6)	16 (19.3)	45 (15.5)	13 (8.3)	3 (4.9)	3 (7.5)	3 (8.6)	22 (7.5)	

### Factors associated with ACEs

Before examining the impact of each independent variable in the model, it is needed to ascertain whether the model enhances our capability to forecast the outcome. The significant chi-square value (p<0.05) suggests a significant enhancement of the Final model compared to the baseline intercept-only model, signifying that the model provides more valuable insights than mere random predictions based on marginal probabilities for the outcome categories (**Table 4**).

**Table 4:** Showing the Model fitting Information, Goodness of fit Test, Pseudo R-Square, and Test of Parallel Lines of both groups.

Model fitting Information					
	Model	-2Log Likelihood	Chi-Square	Df	Sig.
**Schizophrenia group**	Intercept Only Final	789.768 533.735	256.033	25	< 0.001
**patient attendants**	Intercept Only Final	691.250 580.070	111.180	27	0.001
Goodness of fit test					
		Chi-Square	df		Sig.
**Schizophrenia group**	Pearson Deviance	825.763 533.735	842 842		0.649 1.000
**patient attendants**	Pearson Deviance	851.455 575.911	834 834		.330 1.000
Pseudo R-Square					
**Schizophrenia group**	Cox and Snell Nagelkerke McFadden				0.585 0.627 0.324
**patient attendants**	Cox and Snell Nagelkerke McFadden				0.316 0.348 0.160
Test of Parallel Lines					
	Model	-2LogLikelihood	Chi-Square	Df	Sig.
**Schizophrenia group**	Null Hypothesis General	533.735 485.347b	48.388c	50	0.538
**patient attendants**	Null Hypothesis General	580.070 522.817b	57.253c	54	0.355

#### Evaluation of the fitted model

The purpose of these statistics is to determine if the fitted model and the observed data are consistent. It starts from the null hypothesis that the fit is good. If we do not reject this hypothesis (i.e., if the p value is large), we can conclude that the data and the model predictions are similar and have a good model. The results of this analysis show that the model does fit very well for both groups (p > 0.05).Additionally, Nagelkerke’s R was 0.627 for individuals with schizophrenia and 0.348 for the patient attendants, suggesting that 62.7% and 34.8% of the variations in outcome variables were elucidated by the model’s independent variables, while the residual 37.3% and 65.2% were attributed to unexplained factors and errors. On the other hand, McFadden’s pseudo-R2 was 0.324 for people with schizophrenia and 0.16 for the patient attendants, suggesting that 32.4% and 16% of the variations in outcome variables were explained by the model’s explanatory variables, while the remaining 67.6% and 84% were attributed to unexplained factors and errors (**Tables 4**).

#### Evaluation of the of proportional odds model

The evaluation involves the use of the parallel line test to determine conformity with the proportional odds model assumption. If the general model exhibits a substantially superior fit with the data compared to the ordinal (proportional odds) model (i.e., if p<0.05), the assumption of proportional odds is refuted. Conversely, the parallel line test for both group (**Table 4**) displayed a non-significant value (p-value>0.05), leading to the acceptance of the proportional odds assumption.

In the bivariable ordinal logistic analysis, variables such as Age, occupation, prior history of tobacco use, and marital status did not show statistical significance in either group. The multivariable ordinal logistic regression analysis among individuals with schizophrenia (**Table 5**) showed that the first two lower education categories (unable to read and write and able to read and write), attachment-related anxiety, low resilience coping, low and moderate social support, alcohol abstention, social drinking, and suicidal tendencies exhibited statistical significance in association with ACEs.

**Table 5:** Bivariable and multivariable ordinal logistic regression analysis of ACEs among people with schizophrenia, Bahir Dar, Amhara, Ethiopia, 2023.

Variables	Categories	COR (95%CI)	AOR (95%CI)	Estimate (B)	P-value
Sex	Male	0.47 (0.31 - 0.72)	0.68 (0.40 - 1.14)	-.390	0.142
	Female	1	1	0a	.
Residency	Rural	2.40 (1.57 - 3.69)	1.49 (0.89- 2.49)	.396	0.131
	Urban	1	1	0a	.
Marital status	Single	1.33 (0.57 - 3.10)	1.20 (0.43 - 3.40)	.184	0.727
	Married	0.57 (0.25 - 1.31)	0.42 (0.16 - 1.14)	-.872	0.085
	Divorced	1.57 (0.58 - 4.27)	1.48 (0.45 - 4.93)	.393	0.514
	Widowed	1	1	0a	.
Education	Unable to read and write	11.73 (5.65 - 25.02)	4.69 (1.94 - 11.61)	1.545	0.001
	Able to write and read	4.96 (2.47 - 10.11)	2.50 (1.09 - 5.80)	.918	0.030
	Primary (1-8 grade)	1.85 (0.92 - 3.73)	1.11 (0.50 - 2.50)	.106	0.797
	Secondary (9-10 grade)	0.79 (0.42 - 1.49)	0.68 (0.32 - 1.44)	-.385	0.320
	Higher education (diploma and above)	1	1	0a	.
Attachment related avoidance	Low	0.11 (0.06 - 0.18)	0.81 (0.29 - 2.27)	-.217	0.681
	High	1	1	0a	.
Attachment related anxiety	Low	0.12 (0.07 - 0.20)	0.15 (0.60 - 0.39)	-1.869	< 0.001
	High	1	1	0a	.
Brief resilient coping	Low resilient cope	2.60 (1.56 - 4.38)	2.07 (1.11 - 3.90)	.728	0.023
	Medium resilient cope	1.01 (0.62 - 1.67)	0.70 (0.38 - 1.29)	-.354	0.258
	High resilient cope	1	1	0a	.
Social support	*Low social support*	8.39 (5.01 - 14.27)	3.93 (2.13 - 7.32)	1.369	< 0.001
	*Moderate social support*	4.16 (2.34 - 7.47)	3.76 (1.93 - 7.42)	1.325	< 0.001
	*Strong social support*	1	1	0a	.
Alcohol use	Abstainer	0.13 (0.06 - 0.27)	0.25 (0.11- 0.57)	-1.401	0.001
	Social drinker	0.19 (0.10 - 0.37)	0.28 (0.12 - 0.64)	-1.273	0.002
	Hazardous drinker	0.67 (0.32 - 1.40)	0.53 (0.22 - 1.30)	-.628	0.162
	Harmful drinker	0.85 (0.40 - 1.82)	0.79 (0.32 - 1.96)	-.240	0.590
	Probable alcohol dependence	1	1	0a	.
Former chat use	No	0.43 (0.26 - 0.69)	0.73 (0.41 - 1.29)	-.322	0.268
	Yes	1	1	0a	.
Suicide behavior	No	0.21 (0.13 - 0.35)	0.27 (0.14 - 0.49)	-1.321	< 0.001
	Yes	1	1	0a	.
Somatic symptom	No to minimal	0.65 (0.33 - 1.26)	0.92 (0.40 - 2.13)	-.079	0.854
	Low	1.11 (0.56 - 2.21)	0.84 (0.36 - 1.94)	-.180	0.674
	Medium	0.40 (0.19 - 0.83)	0.63 (0.26 - 1.52)	-.464	0.305
	High	0.60 (0.29 - 1.21)	0.57 (0.24 - 1.35)	-.561	0.201
	Very high	1	1	0a	.

AOR, adjusted odd ratio; COR, crud odd ratio; OR, Odd ratio; CI: 95% confidence interval for coefficients. 1 refers to the reference category OR Positive coefficients tell us that higher values of the explanatory variable are associated with higher outcome, while negative coefficient tell us that higher value of the explanatory variable are associated with lower outcome.

The likelihood of experiencing four or more ACEs was 4.7 times (AOR, 95% CI: 1.94 -11.61) and 2.5 times (AOR, 95% CI: 1.09 - 5.79) higher among people with schizophrenia who are unable to read and write, and able to read and write, respectively, compared to those with higher education. The probability of having four or more ACEs was 84.6% lower (AOR, 95% CI: 0.59–0.39) among people with schizophrenia exhibiting low attachment-related anxiety compared to those with high attachment-related anxiety. The probability of experiencing four or more ACEs among individuals with schizophrenia with poor social support was 3.93 times higher (AOR, 95% CI: 2.13 - 7.32) than those with strong social support.

The multivariable ordinal logistic regression analysis conducted in the patient attendants (**Table 6**) revealed significant associations between being unable to read and write, attachment-related anxiety, rural residency, and ACEs. The analysis indicated that rural residents in the patient attendants were 1.73 times more likely to report four or more ACEs compared to urban residents (AOR, 95% CI: 1.05–2.85).

**Table 6:** Bivariable and multivariable ordinal logistic regression analysis of ACEs among patient attendants, Bahir Dar, Amhara, Ethiopia, 2023.

Variables	Categories	COR (95%CI)	AOR (95%CI)	Estimate (B)	P-value
Sex	Male	0.61(0.40 - 0.94)	0.92 (0.55 -1 .54)	-0.083	0.754
	Female	1	1	0^a^	.
Residency	Rural	1.95 (1.28 - 3.00)	1.73 (1.05 - 2.85)	0.549	< 0.031
	Urban	1	1	0^a^	.
Education	Unable to read and write	4.59 (2.16 - 9.95)	3.00 (1.30 - 7.00)	1.099	< 0.011
	Able to write and read	1.03 (0.53 - 1.99)	0.92(0.43 -1.98)	-0.086	0.824
	Primary (1-8 grade)	1.18 (0.63 - 2.21)	0.93 (0.46 - 1.90)	-0.071	0.845
	Secondary (9-10 grade)	1.11 (0.60 - 2.03)	0.70(0.35 - 1.42)	-0.352	0.321
	Higher education	1	1	0^a^	.
Attachment related avoidance	Low	0.18 (0.11 - 0.30)	0.55(0.22- 1.24)	-0.645	0.142
	High	1	1	0^a^	.
Attachment related anxiety	Low	0.11 (0.060 - 0.19)	0.20 (0.08 - 0.50)	-1.592	< 0.001
	High	1	1	0^a^	.
Brief Resilient Coping	Low resilient cope	4.93 (2.55 - 9.67)	0.88 (0.32- 1.42)	-0.129	0.803
	Medium resilient cope	1.33 (0.83 - 2.12)	1.49 (0.76 -2.90)	0.395	0.247
	High resilient cope	1	1	0^a^	.
Social support	*Low social support*	4.60 (2.40 - 8.94)	1.91 (0.71-5.12)	0.645	0.202
	*Moderate social support*	1.32 (0.83 - 2.10)	0.85 (0.44 - 1.66)	-0.160	0.639
	*Strong social support*	1	1	0^a^	.
Former chat use	No	0.40 (0.20 - 0.77)	0 .57 (0.27- 1.20)	-0.569	0.136
	Yes	1	1	0^a^	.
Suicide behaviors	No	0.43 (0.20 - 0.92)	0.75 (0.32- 1.74)	-0.294	0.495
	Yes	1	1	0^a^	.

AOR, adjusted odd ratio; COR, crud odd ratio; OR, Odd ratio; CI: 95% confidence interval for coefficients.

1 refers to the reference category OR.

a. Set to zero because this parameter is redundant.

## Discussions

This study compared a group of 293 healthy controls of the patient attendants with 291 individuals diagnosed with schizophrenia to assess the prevalence of ACEs. In the current study, results showed that 46.8% of the patient attendants and 69.4% of individuals with schizophrenia reported at least one ACE, lower than rates in South Africa and Argentina (94% and 87% respectively) (50, 61). This discrepancy may be due to variations in measurement tools such as the Childhood Trauma Questionnaire (CTQ) used in previous studies.

People with schizophrenia had higher rates of at least one ACE and four or more ACEs compared to the patient attendants group (69.4% and 28.5% vs. 46.8% and 11.9% respectively). Notably, the study found that schizophrenia patients were over twice as likely to report four or more ACEs compared to patient attendants participants. These results align with previous studies indicating a higher prevalence of ACEs among individuals with schizophrenia, supporting the trauma multiplicity theory. The dose-response model suggests that accumulating multiple ACEs increases the risk of developing schizophrenia (62–64).

While ACEs are doubtless associated with poorer mental health, there are also plenty of healthy individuals with several ACEs but without any mental problems. The reason why all people who had ACEs do not develop schizophrenia indicates that schizophrenia is considered to be a biological (65–67). “A Neural Diathesis-Stress Model of Schizophrenia” Reiterate the commonly held belief that stressors can exacerbate symptoms but do not constitute causative factors. Cite standard research suggesting heightened susceptibility to stressors is linked to schizophrenia vulnerability. ACEs are considered relevant only to the extent that they exacerbate premorbid behavioral dysfunction or, at most, accelerate the onset of the initial clinical episode (67). The reason why some individuals without ACEs also have schizophrenia is that there exists a model that suggests that factors such as ACEs are not the only ones that determine a person’s susceptibility to symptoms of schizophrenia. The first is the multiple etiological hypotheses of schizophrenia that are in use today (68). Our intention was related to the models that suggest examining with an open mind and conducting appropriate research to determine whether adverse childhood events could be a contributing factor, either alone or in combination with the effects of genetic risk or prenatal factors (69).

In terms of health-related risk factors, the study revealed that individuals with schizophrenia and low social support had nearly four times higher odds of reporting four or more ACEs compared to those with strong social support (AOR = 3.93, 95% CI: 2.13 - 7.32). Additionally, individuals with medium social support had 3.76 times higher odds of experiencing four or more ACEs. This underscores the association between low social support and ACEs among individuals with schizophrenia, highlighting the potential benefits of social support interventions in preventing psychosis development in individuals with a history of child maltreatment (70).

Suicidal behaviors in people with schizophrenia have been linked to a history of multiple ACEs, a connection not observed in the patient attendants. Individuals with schizophrenia who exhibited suicidal behaviors were 3.75 times more likely to report four or more ACEs compared to those without such behaviors, a correlation also noted in previous research (27, 71). These chronically ACEs may impact the hypothalamic pituitary adrenal axis, leading to brain changes that increase susceptibility to stress in adulthood (26).

In the context of schizophrenia, people with low resilient coping skills are 2.07 times more likely to have four or more ACEs compared to those with high resilient coping skills, aligning with findings that childhood emotional and sexual abuse are linked to passive and avoidance coping in those with psychotic disorders (41).

Among people with schizophrenia, the odds of reporting four or more ACEs were 75% lower for alcohol abstainers (AOR = 0.25, 95% CI: 0.11 - 57) and 72% lower for social drinkers (AOR = 0.28, 95% CI: 0.12 - 0.64) when compared to those with probable alcohol dependence. This difference could be attributed to ACEs heightening the risk of psychosis development by hindering the maturation of adaptive emotion regulation mechanisms and fostering maladaptive coping strategies (40).

People with schizophrenia experiencing low attachment-related anxiety are 85% (AOR = 0.15, 95% CI: 0.59–0.39) less likely to have four or more ACEs than those with high attachment-related anxiety, consistent with evidence suggesting that disrupted attachment plays a role in connecting childhood trauma to adult psychotic symptoms (72, 73). Regarding education, individuals with less than primary school education showed a notable increase in the likelihood of having four or more ACEs, while those with primary or high school education did not exhibit a significant rise compared to those with higher education, in line with research findings (74).

## Strengths and limitations

The study has several strengths. First, the data were collected by mental health professionals proficient in clinical interviews, enhancing data reliability in a cost-effective manner. Second, we approached ACEs as an ordinal outcome variable, enabling an assessment of category frequency between population groups. The inference of ACEs prevalence in individuals with schizophrenia and the patient attendants depends on observing each category, rather than just the presence or absence of ACEs. This method also reveals the correlation between ACEs and the independent variable as ACEs increase exponentially, ranging from low to high. Compared to multinomial logistic regression or binary logistic regression, this approach reduces information loss between categories, affecting the drawn conclusions. However, limitations of the study include the retrospective data collection method, which introduces risks of recall bias, and the potential influence of defense mechanisms on recollection was not accounted for. The data collection process was interviewer administered and focused on sensitive topics, possibly prompting respondents to provide socially desirable responses. The use of patient attendants as control might not representative of the general population because it might inflate the rate of reported ACEs. The other thing, we did not adjust the significance value for multiple testing and that certain tests would therefore not survive a stricter alpha criterion. In the long run, we acknowledge that the study’s shortcoming was that we did not examine the relationship between ACEs and clinical characteristics such as the amount of medications taken, the length of treatment, and the type or severity of symptoms.

## Conclusion and recommendation

ACEs, although reported by the patient attendants, are depicted as being more abundant, widespread, and significantly associated with a higher number of health-related risk factors in people with schizophrenia. Recognizing the multiplicity and interconnectedness of these factors could prove beneficial for psycho-social interventions. Further research is warranted to explore which relational and individual protective variables contribute to the resilience exhibited by some adults who have faced ACEs without developing psychopathology. The extensive correlation between ACEs and health-related risk factors underscores the critical importance of primary prevention strategies to mitigate the occurrence of ACEs.

## Data availability statement

The raw data supporting the conclusions of this article will be made available by the authors, without undue reservation.

## Ethics statement

The studies involving humans were approved by Bahir Dar University College of Medical and Health Science Institutional Review Board. The studies were conducted in accordance with the local legislation and institutional requirements. The participants provided their written informed consent to participate in this study.

## Author contributions

BA: Conceptualization, Data curation, Formal analysis, Funding acquisition, Investigation, Methodology, Project administration, Resources, Software, Supervision, Validation, Visualization, Writing – original draft, Writing – review & editing. TBel: Conceptualization, Data curation, Investigation, Methodology, Resources, Software, Supervision, Visualization, Writing – review & editing. RD: Conceptualization, Data curation, Formal analysis, Investigation, Methodology, Resources, Software, Supervision, Visualization, Writing – review & editing, Writing – original draft. TBet: Conceptualization, Formal analysis, Investigation, Writing – review & editing. SM: Conceptualization, Data curation, Formal analysis, Investigation, Methodology, Software, Supervision, Writing – review & editing.

## Glossary

**Table:**

ACEs	Adverse Childhood Experiences
ACE-Q	Adverse childhood experience - Questioner
AOR	Adjusted Odds Ratio
AUDIT	Alcohol Use Disorders Identification Test
CI	Confidence Interval
CT Q-SF	Childhood Trauma Questioner -Short-Form
DSM-5	Diagnostic and Statistical Manual of Mental Disorders the Fifth Edition
ECR-RS	Experiences in Close Relationships - Relationship Structures
FHCSH	Felege Hiwot Comprehensive Specialized Hospital
OR	Odds Ratio
P-value	Probability Value
SBQ-R	Suicide Behaviors Questionnaire - Revised
SPSS	Statistical Package for Social Science
SSD	Schizophrenia Spectrum Disorder
SSS-8	Somatic Symptom Scale - 8
TGCSH	Tibebe Ghion Comprehensive Specialized Hospital
